# Autophagy Constitutes a Protective Mechanism against Ethanol Toxicity in Mouse Astrocytes and Neurons

**DOI:** 10.1371/journal.pone.0153097

**Published:** 2016-04-12

**Authors:** Antoni Pla, María Pascual, Consuelo Guerri

**Affiliations:** Department of Cellular Pathology, Centro de Investigación Príncipe Felipe, C/ Eduardo Primo Yúfera 3, 46012, Valencia, Spain; Centre national de la recherche scientifique, University of Bordeaux, FRANCE

## Abstract

Ethanol induces brain damage and neurodegeneration by triggering inflammatory processes in glial cells through activation of Toll-like receptor 4 (TLR4) signaling. Recent evidence indicates the role of protein degradation pathways in neurodegeneration and alcoholic liver disease, but how these processes affect the brain remains elusive. We have demonstrated that chronic ethanol consumption impairs proteolytic pathways in mouse brain, and the immune response mediated by TLR4 receptors participates in these dysfunctions. We evaluate the *in vitro* effects of an acute ethanol dose on the autophagy-lysosome pathway (ALP) on WT and TLR4^-/-^ mouse astrocytes and neurons in primary culture, and how these changes affect cell survival. Our results show that ethanol induces overexpression of several autophagy markers (ATG12, LC3-II, CTSB), and increases the number of lysosomes in WT astrocytes, effects accompanied by a basification of lysosomal pH and by lowered phosphorylation levels of autophagy inhibitor mTOR, along with activation of complexes beclin-1 and ULK1. Notably, we found only minor changes between control and ethanol-treated TLR4^-/-^ mouse astroglial cells. Ethanol also triggers the expression of the inflammatory mediators iNOS and COX-2, but induces astroglial death only slightly. Blocking autophagy by using specific inhibitors increases both inflammation and cell death. Conversely, in neurons, ethanol down-regulates the autophagy pathway and triggers cell death, which is partially recovered by using autophagy enhancers. These results support the protective role of the ALP against ethanol-induced astroglial cell damage in a TLR4-dependent manner, and provide new insight into the mechanisms that underlie ethanol-induced brain damage and are neuronal sensitive to the ethanol effects.

## Introduction

Autophagy is a catabolic process that involves the degradation of cytoplasmic components through an autophagosome-lysosome pathway (ALP) by facilitating the removal of superfluous and damaged organelles to help cells adapt to changing nutrient conditions and maintain their homeostasis. This proteolytic pathway plays a crucial role in the physiology and pathology of the central nervous system [1], and is critical in neuronal homeostasis since neurons are post-mitotic cells and require effective protein degradation to prevent accumulation of toxic aggregates. Nevertheless, dysfunction of autophagy causes accumulation of abnormal proteins and/or damaged organelles, which leads to synaptic dysfunction, cellular stress, and neuronal and glial cell death. Abnormal autophagy is involved in the pathology of both neurodegenerative disorders, such as Alzheimer’s, Parkinson’s, Huntington’s disease [2], as well as acute brain injuries [1].

During autophagy, different proteins intervene to regulate the distinct steps of the process. One of the most important set of molecules to regulate autophagy is ATG proteins (“Autophagy-related”). These proteins associate and form complexes that control the sequential steps of autophagy, like ULK1/ATG1 in the induction process, beclin-1/ATG6 in membrane nucleation, or ATG5, ATG12 and LC3/ATG8 in the vesicular elongation of the phagophore [3]. On top of that, upstream of these ATG proteins, the mammalian target of rapamycin (mTOR) constitutes the main negative regulator of autophagy, and represses it in the presence of growth factor and nutrients [4].

We have recently showed that chronic ethanol consumption is capable of altering proteolytic pathways, including autophagy, in the mouse brain [5]. Alcohol is a neurotoxic compound whose abuse can cause neural damage, protein oxidation, and even neurodegeneration [6]. Ethanol activates innate immune system receptor TLR4 in glial cells to induce gliosis, production of cytokines and neuroinflammation [7,8]. Interestingly, we have demonstrated that mice chronically fed with ethanol show impaired autophagy, and display low levels of the main ATG proteins (ATG5, ATG12, LC3-II), possibly caused by an up-regulation in the phosphorylation levels of mTOR [5]. In fact, autophagy has been in the spotlight in many neurodegenerative and inflammatory diseases, and many of these pathologies are characterized by autophagic dysfunctions, although it is still unclear if these are actually the cause or the consequence of the pathology itself. The use of autophagy modulators, such as rapamycin or wortmannin (inducer and inhibitor, respectively), is frequent in the treatment of these diseases [9]. However, the effects of alcohol on autophagy are still controversial, and may depend on dosage, consumption type or the organ affected. Whereas some studies state that ethanol impairs autophagic flux [10,11], other works support the idea that activation of autophagy could be a protective mechanism against ethanol-induced cell death [12]. At the same time, whether autophagy is differentially induced in neurons or astroglial cells treated with ethanol, and the role of autophagy with regard to ethanol-induced cell death, remain unclear. In fact, astrocyte function impairment could lead to neuronal dysfunction which could contribute to the pathology of the disease.

Herein we report that a single ethanol dose (50 mM) enhances the expression of different autophagic proteins (e.g., ULK1/ATG1, beclin-1/ATG6, ATG12 and LC3/ATG8), and increases the number of autophagosomes and lysosomes in cultured astrocytes, possibly through the modulation of autophagy regulator mTOR. Inhibition of autophagy not only increases the ethanol-induced inflammatory mediators such as iNOS and COX-2, but also enhances apoptotic and necrotic astrocytic death. We also provide evidence that neurons and astrocytes respond differently as ethanol down-regulates the autophagy pathway and triggers cell death in neurons, and that these effects are partially recovered by using autophagy enhancers. Our results also support the role of the TLR4 response since ethanol does not alter the ALP in astroglial cells of TLR4-KO mice. These findings provide new insights of the differential role of autophagy in ethanol-induced cell death in neurons and astrocytes, and highlight the role of proteolytic pathways and TLR4 response in ethanol-induced neural damage.

## Materials and Methods

### Animals

C57BL/6 wild-type (WT) mice (Harlan Ibérica, Barcelona, Spain) and TLR4-KO knockout (KO) mice (C57BL/6 background, kindly provided by Dr. S. Akira, Osaka University, Suita, Japan) of both sexes were used. All the animals were kept under controlled light and dark conditions (12/12 h), temperature (23°C), and humidity (60%). After mating, dams were placed in separate cages during the gestation period, and then the fetuses (17-day-old) and newborn mice were sacrificed by decapitation to perform neuronal and astroglial cultures, respectively. All the experimental procedures were carried out in accordance with the guidelines approved by the European Communities Council Directive (86/609/ECC) and by Spanish Royal Decree 1201/2005. The animal experiments were also approved by the *Ethical Committee of Animal Experimentation* of the Príncipe Felipe Research Center (Valencia, Spain).

### Culture of astroglial cells and ethanol treatment

Cerebral cortices from newborn mice of both sexes were dissected, carefully stripped of their meninges and mechanically dissociated in Dulbecco’s modified Eagle’s medium (DMEM) (Gibco-Life Technologies, Madrid, Spain). The mixed cell suspension of both sexes was vortexed at maximum speed (1 min) and filtered through a nylon mesh (80-μm pore size). Cells were plated on 55-mm Nunc plastic tissue culture dishes (850 cells/mm^2^) and maintained in DMEM that contained 20% foetal bovine serum (FBS) supplemented with L-glutamine (1%), glucose (1%), fungizone (1%) and antibiotics (1%) (Gibco-Life Technologies). Cultures were grown in a humidified atmosphere of 5% CO_2_/95% air at 37°C. After 1 week of culture, FBS was reduced to 10%, glucose was removed, and the medium was changed twice a week. Cells were grown to confluence and were used after 12 days in culture. The culture purity of the astroglial cells was assessed by immunofluorescence, as previously described [13].

To assess the effects of ethanol (50 mM) on astroglial cells, this compound was added to DMEM in the presence of a low concentration of FBS (2%), since serum starvation induces autophagy in many cells [14], including astrocytes (data not shown). At 0, 0.5, 1, 3, 7 and 24 h of ethanol treatment, cells were harvested by trypsinization, centrifuged and used for specific determinations.

### Culture of neurons and ethanol treatment

Cerebral cortices from 17-day-old fetal wild-type mice of both sexes were dissected, carefully stripped of their meninges and mechanically dissociated in Hank’s Balanced Salt Solution (HBSS) (Gibco-Life Technologies). The mixed cell suspension of both sexes was filtered through a nylon mesh (80-μm pore size) and centrifuged at 200 g for 3 minutes. The remaining pellet was resuspended in Neurobasal medium that contained 2% B-27 supplement with GlutaMAX (1%), L-glutamine (1%), fungizone (1%) and antibiotics (1%), and cells were plated on 55-mm Nunc plastic tissue culture dishes (850 cells/mm^2^) treated with Poli-D-Lysine (12.5 μg/ml) (all from Gibco-Life Technologies). Cultures were grown in a humidified atmosphere of 5% CO_2_/95% air at 37°C. Cells were grown to confluence and were used after 7 days in culture.

To assess the effects of ethanol (50 mM) on neurons, this compound was added to Neurobasal medium. At 0, 3, 7 and 24 h of ethanol treatment, cells were harvested by trypsinization, centrifuged and used for specific determinations. In some experiments, the medium of neuron, at day 5 in culture, was removed and replaced with a conditioned medium from astroglial cells treated with or without ethanol (50 mM) for 24 h. Neurons were incubated with astroglia-conditioned medium for 1 day. Then cells were harvested and used for specific determinations. Alcohol determinations in the medium were assessed with a spectrophotometric assay kit (Sigma-Aldrich, Madrid, Spain).

### Western blot analysis

Cells were harvested in PBS, centrifuged and then lysed in lysis buffer (1% Nonidet P-40, 20 mM Tris-HCl, pH 8, 4 mM sodium chloride, 40 mM sodium fluoride and protease inhibitors) for 30 min on ice. An equal amount of cell lysate of each sample (40 μg of protein/lane) was loaded onto sodium dodecyl sulphate-polyacrylamide gels (SDS-PAGE), and was then blotted onto polyvinylidene fluoride membranes. Membranes were blocked with 5% BSA in TBS that contained 0.1% Tween-20 (TBS/T), and were then incubated overnight with the following primary antibodies: anti-APG5L/ATG5, anti-LC3A/B, anti-SQSTM1/p62, anti-p-ULK1 and anti-ULK1 (Abcam, Cambridge, UK); anti-ATG12, anti-Cathepsin B, anti-p-mTOR and anti-mTOR (Santa Cruz Biotechnology, Heidelberg, Germany); anti-caspase 3 (active fragment of 17 kDa), anti-p-beclin-1 (Cell Signaling, Massachusetts, USA); anti-beclin-1 (Abgent, California, USA); anti-iNOS (BD Transduction Laboratories, California, USA) and anti-COX-2 (Cayman Chemical, Michigan, USA). After washing with TBS/T, blots were incubated with HRP-conjugated antibodies. Blots were done using the ECL system (ECL Plus; Fisher Scientific, Madrid, Spain). All the membranes were stripped for 30 min in sodium dodecyl sulfate (SDS) solution (0.4% SDS and 200 mM glycine, pH 2.5), and were washed and incubated with anti-GAPDH mAb (Chemicon, California, USA) or with the corresponding total form of the phosphorylated protein as a loading control. The intensity of the bands was quantified with the ImageJ image analysis software (version 1.44p, National Institutes of Health, Bethesda, USA). The densitometry analysis is shown in arbitrary units normalized to the loading control. The densitometric analysis of the basal levels of the different proteins evaluated in WT and TLR4-KO astrocytes is included in S1 Table (Supporting Information).

### Immunofluorescence

For the immunofluorescence studies, astroglial cells were plated on 15-mm glass coverslips in 12-well culture plates. Cells, either treated with or without ethanol (50 mM) for 3 and 24 h, were fixed with 3.7% paraformaldehyde in PBS (with Ca^2+^ and Mg^2+^) for 20 min, and permeabilized with 0.25% Triton X-100 for 5 min. Cells were then incubated with anti-cathepsin B polyclonal antibody (1:50, Santa Cruz Biotechnology) and anti-GFAP polyclonal antibody (1:50, Santa Cruz Biotechnology). Incubation was carried out overnight at 4°C. After several washings in PBS, cells were incubated for 1.5 h at 37°C with the corresponding Alexa Fluor-conjugated antibodies (1:500, Sigma-Aldrich). Nuclei were stained with 0.5 μg/mL Hoechst 33342 dye (Molecular Probes, Madrid, Spain). Coverslips were mounted in FA mounting fluid (Difco, Madrid, Spain). Fluorescence images were analyzed in single cells using a Leica confocal microscope (model TCS-SP2-AOBS, Mannheim, Germany). Fluorescence was quantified with the ImageJ software, and the results were expressed as the threshold/cell (arbitrary units).

### Electron microscopy

Astrocytes treated with or without ethanol were fixed with 2.5% glutaraldehyde in 0.1 M phosphate buffer (pH 7.4) for 5 min at 37°C. Then a new solution of 2.5% glutaraldehyde was added for 1–2 h at 4°C. Following several washes with 0.1 M phosphate buffer, cells were post-fixed with 2% osmium, rinsed, dehydrated and embedded in Durcupan resin (Sigma-Aldrich). Semithin sections (1.5 μm) were cut with an Ultracut UC-6 (Leica, Heidelberg, Germany) and stained lightly with 1% toluidine blue. Finally, ultra-thin sections (0.08 μm) were cut with a diamond knife, stained with lead citrate (Reynolds solution) and examined under a transmission electron microscope FEI Tecnai G2 Spirit (FEI Europe, Eindhoven, Netherlands) using Morada a digital camera (Olympus Soft Image Solutions GmbH, Münster, Germany). The number of lysosomes was calculated from 20 to 25 micrographs from each sample of at least three cell cultures per group. The results were expressed as the number of lysosomes/cell.

### Lysosomal pH measurements

Astroglial cells, either treated with or without ethanol (50 mM) for 3 and 24 h, in the presence of 2% FBS, were incubated with 1 mg/ml FITC-dextran (40 kDa, Sigma-Aldrich) for 18 h. Then cells were washed twice with PBS and chased for 4 h with the corresponding fresh medium in the presence or absence of ethanol, but without FITC-dextran. We used a standard calibration curve of several pHs within the 4.0–8.0 range. To allow the lysosomal pH to match the medium pH, some cells were treated with 10 μM nigericin and 20 μM monensin before the flow cytometry analysis (Sigma-Aldrich). Samples were analyzed with a Cytomics FC500 flow cytometer (Beckman Coulter, Barcelona, Spain) using excitation and emission fluorescence of 488±28 nm and 530±28 (FL1)-610±20 (FL3) nm, respectively. Fluorescence values were linearized *vs* pH and plotted in each calibration curve. The lysosomal pH of astrocytes was calculated by interpolating the mean fluorescence intensity of each treatment on its respective calibration curve.

### Measurement of lysosomal content

Astroglial cells, either treated with or without ethanol (50 mM) for 3 and 24 h in the presence of 2% FBS, were seeded in 12-well plates. All the cells were incubated with 50 nM Lysotracker Red DND-99 (Molecular Probes, Madrid, Spain) for the last 2 h of ethanol treatment. Next cells were washed and fluorescence was analyzed with a Cytomics FC500 flow cytometer (Beckman Coulter). These data were corroborated by immunofluorescence studies after incubation with 200 nM Lysotracker Red DND-99 for 2 h. The inmunofluorescence procedure was carried out as described above.

### Autophagy modulation and cell death quantification by flow cytometry

Astrocytes and neurons from WT mice were incubated for 24 h either with or without ethanol (50 mM) in the presence or absence of the different autophagy modulators: wortmannin (100 nM, MP Biomedicals, California, USA) and bafilomycin A1 (100 nM, Santa Cruz Biotechnology) as inhibitors, and rapamycin (100 nM, LC Laboratories, Massachusetts, USA) as an inducer. The autophagy modulators were incubated in cells 1 h before initiating the 24 h ethanol treatment.

To analyze cell death and to differentiate between apoptosis and necrosis, cells were harvested and incubated with FITC-conjugated annexin V (Sigma-Aldrich) in combination with cell-impermeant DNA fluorophore propidium iodide (PI) (Immunostep, Salamanca, Spain) at room temperature for 15 min. Finally, samples were analyzed with a Cytomics FC500 flow cytometer (Beckman Coulter) and data were illustrated by the CXP software.

### Lactate dehydrogenase activity

The release of lactate dehydrogenase (LDH) in the culture supernatant of astrocytes or neurons was measured using the colorimetric Cyto-Tox 96 Non-radioactive Assay (Promega, Madrid, Spain) following the manufacturer’s instructions. This assay is a coupled enzymatic assay, which results in the conversion of a tetrazolium salt into a formazan product, which in turn is proportional to the number of non-viable cells (cells with a damaged membrane). We used triton X-100 as a positive control. The LDH values were expressed as LDH release rate.

### Statistical analysis

The results are reported as mean ± SEM. Statistical significance for some western blots, biochemical and microscopy studies were determined by an unpaired Student’s t-test. Time course experiments were analyzed using a one-way ANOVA, followed by Dunnett’s *post hoc* test. The experiments in which autophagy inhibitors were used were analyzed by one-way ANOVA, followed by the Newman-Keuls *post hoc* test.

## Results

### Ethanol induces the expression of several proteins of the autophagy machinery in cortical astrocytes: role of TLR4

**Fig 1:**
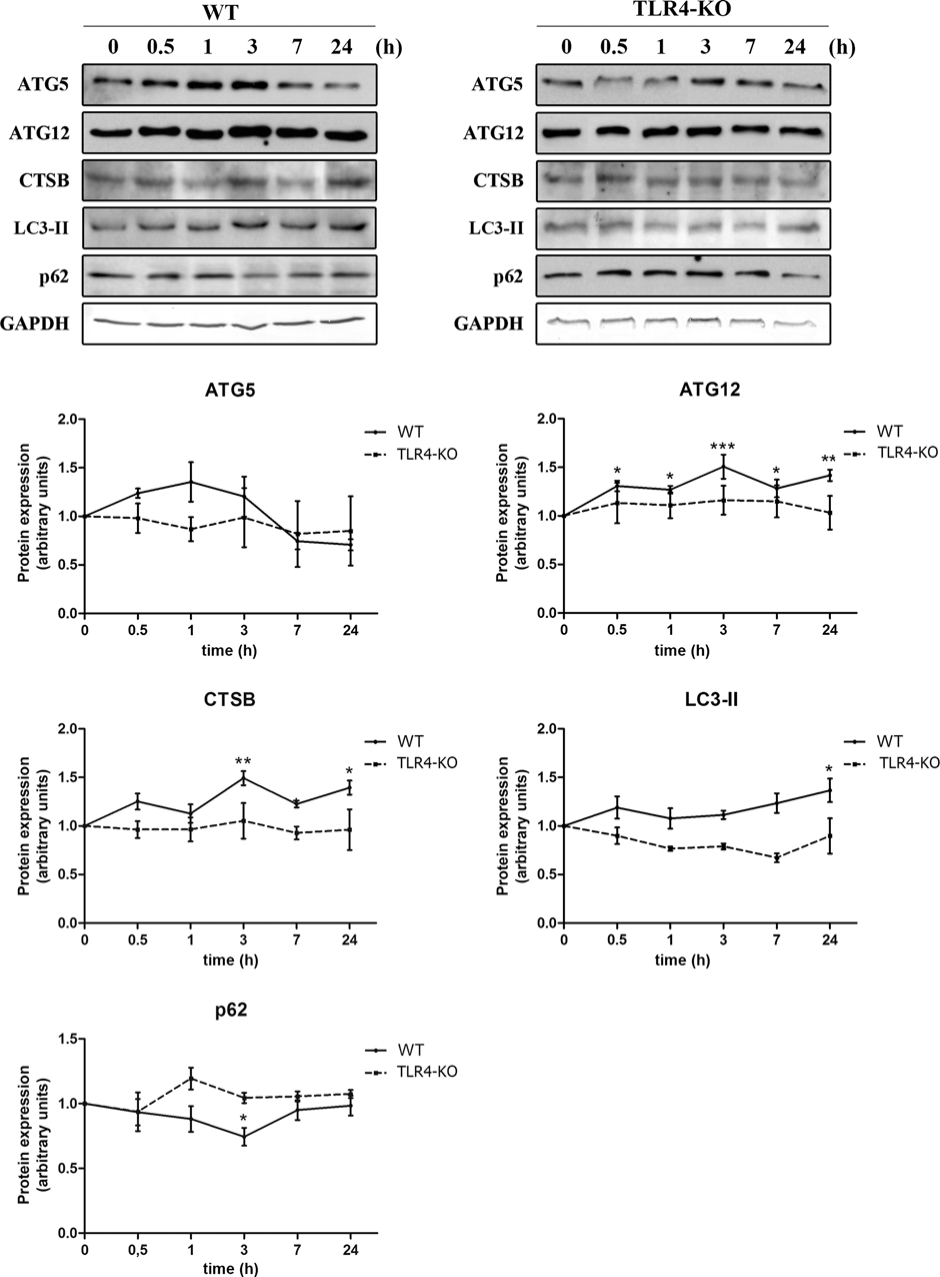
TLR4 participates in the ethanol-induced overexpression of several autophagic proteins in cortical astroglial cells. Immunoblot analysis and quantification of ATG5, ATG12, cathepsin B, LC3-II and p62 in cell extracts of ethanol (50 mM)-treated cells at different time points (0, 0.5, 1, 3, 7 and 24 h). Values represent mean ± SEM, n = 12–15 independent experiments. * p < 0.05, ** p < 0.01, *** p < 0.001 compared with the untreated WT or TLR4-KO value. Blots were stripped, and the total quantity of GAPDH was also assessed. A representative immunoblot of each protein is shown.

To study the ALP we first analyzed whether ethanol was able to alter the expression of several autophagic proteins in cortical astrocytes in culture. Proteins LC3-II and ATG12 participate in the conjugation pathway that is involved in the elongation of the phagophore that will finally form the autophagosome [15]. Ethanol treatment increased the expression of both LC3-II and ATG12, but while the LC3-II levels significantly raised at 24 h post-treatment, ATG12 expression was up-regulated at all time points studied (Fig 1). Additionally, the expression of the p62 protein decreased 3 h after ethanol treatment. This protein can bind to LC3-II, accumulates in the cell under low autophagic activity conditions and is rapidly degraded together with the cargo in autophagolysosomes [16]. It is important to note that we have only measured the levels of LC3-II, since due to the high expression of LC3-I in brain tisues, it is more reliable than the simple comparison of LC3-I and LC3-II, or summation of LC3-I and LC3-II for ratio determinations [17,18]. Another crucial protein in the autophagy pathway is cathepsin B, which is the major lysosomal enzyme required for the degradation of contents in the autophagolysosome. Cathepsin B expression was increased at 3 h and 24 h after ethanol treatment. Interestingly, ethanol induced no significant changes in the time-course experiments with TLR4-KO cells (Fig 1), which indicates the participation of TLR4 in the overexpression of autophagic proteins. In addition, we obtained similar results using primary cultures of microglial cells exposed to 50 mM ethanol (overexpression of LC3-II, ATG12, cathepsin B and a tendency towards p62 down-regulation in WT microglia, and no changes in TLR4-KO cells) to those observed in astrocytes (see S1 Fig in Supporting Information).

### TLR4 participates in the ethanol-induced changes of lysosomal mass and lysosomal pH in cortical astrocytes

**Fig 2:**
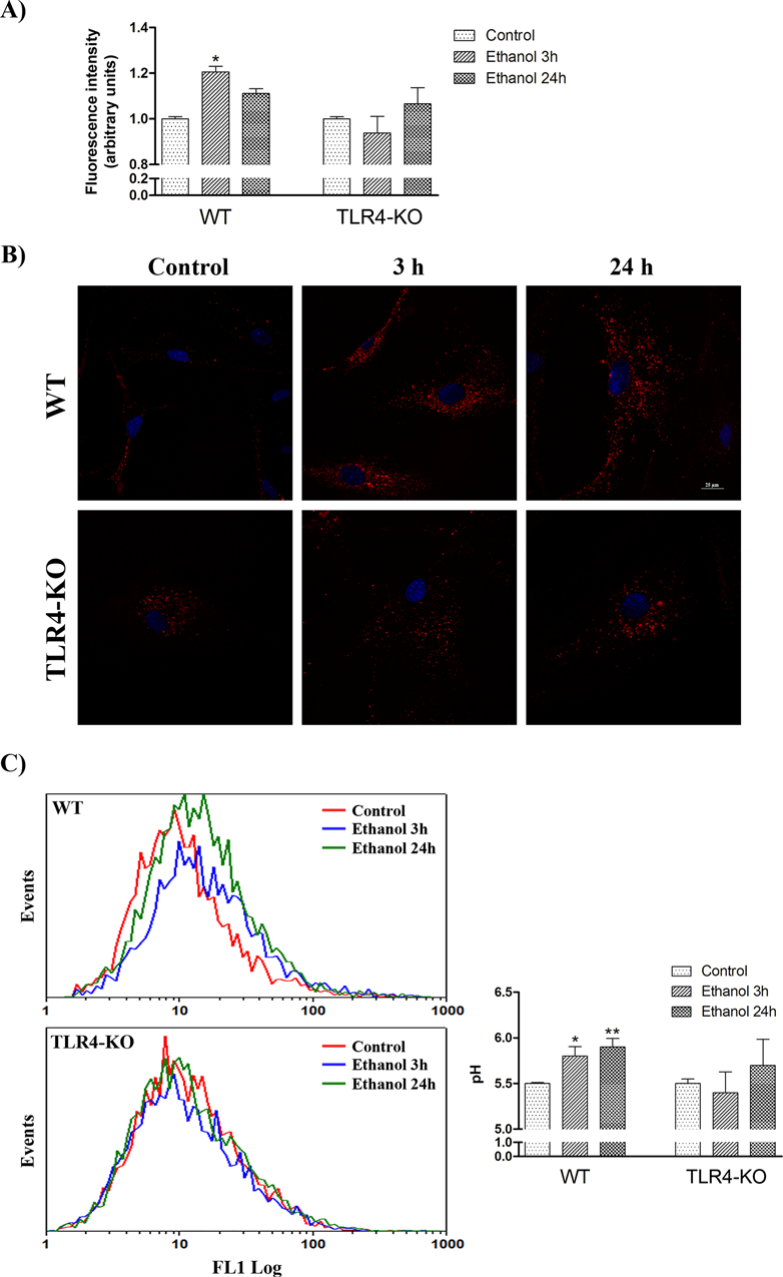
TLR4 contributes to the ethanol-induced changes of lysosomal mass and lysosomal pH in cortical astrocytes. (A) Lysosomal mass was analyzed with the LysoTracker Red fluorescence probe (50 nM) in the WT and TLR4-KO astrocytes treated with or without ethanol for 3 and 24 h. LysoTracker Red fluorescence was analyzed by quantitative flow cytometry. Data represent mean ± SEM, n = 6–7 independent experiments. * p < 0.05 as compared to the untreated WT astrocytes. (B) Immunofluorescence study of LysoTracker Red (200 nM) in the WT and TLR4-KO astrocytes treated with or without ethanol for 3 and 24 h. Scale bar represents 25 μm. (C) Lysosomal pH was analyzed with FITC-dextran (1 mg/ml) by flow cytometry in the WT and TLR4-KO astrocytes treated with or without ethanol for 3 and 24 h. Data represent mean ± SEM, n = 6–7 independent experiments. * p < 0.05, ** p < 0.01 as compared to the untreated WT astrocytes.

**Fig 3:**
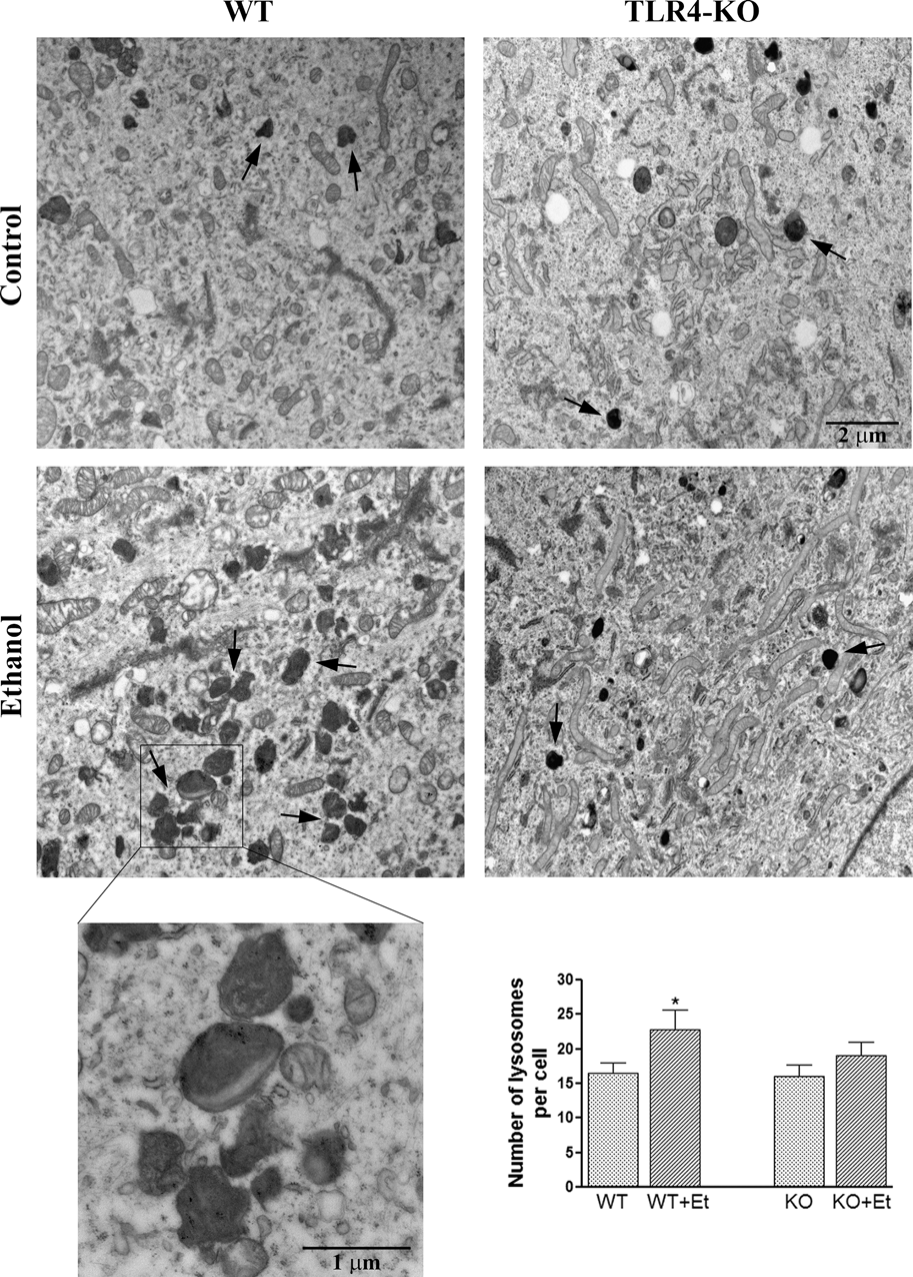
The electron microscopy analysis shows that ethanol treatment increases the number of lysosomes in the WT astrocytes. Representative transmission electron micrographs of the cortical WT and TLR4-KO astrocytes in the culture treated with or without ethanol for 24 h are shown. Arrows indicate lysosomes. Data represent mean ± SEM, n = 3–4 independent experiments.* p < 0.05 as compared to the untreated WT astrocytes.

Autophagosomes fuse with lysosomes to form autophagolysosomes (or simply, autolysosomes) with an acidic pH [19]. Since the lysosomal mass can be estimated by the fluorescence emitted from acidophilic lysosomotropic probe LysoTracker Red, we analyzed by flow cytometry whether ethanol was able to induce changes in lysosomal mass in WT and TLR4-KO astrocytes. Fig 2A shows that ethanol significantly increased the lysosomal mass at 3 h after ethanol treatment in WT astrocytes, and despite a partial recovery, this tendency towards up-regulation was maintained after 24 h of ethanol treatment. The lysosomal mass of the ethanol-treated TLR4-KO astrocytes illustrated no changes as compared with their control counterparts. Furthermore, these data were corroborated by fluorescence microscopy, as shown in Fig 2B. In addition, the ultrastructural analysis of the autophagic machinery (by electron microscopy) revealed that ethanol treatment increased the formation of electron-dense lysosomes in WT astrocytes, whereas the ethanol-treated astrocytes from TLR4-KO mice showed no changes when compared with their control counterparts (Fig 3).

Since the lysosomal degradation capacity and autophagosome maturation are dependent on lysosomal pH [20], we decided to evaluate whether ethanol was able to induce changes in the lysosomal pH of the WT and TLR4-KO astrocytes. For this purpose, cells were incubated with FITC-dextran, a compound that can bind to acidic structures. Fig 2C demonstrates that the lysosomal pH values were slightly higher in ethanol-treated WT astrocytes compared to control WT cells. In contrast, no changes were observed in ethanol-treated TLR4-KO cells. The higher levels of lysosomal pH in the ethanol-treated WT cells could either be associated with an increased uptake of cytosolic material, since the cytosolic material has a neutral pH [21], or it could be indicative of some direct damage caused by ethanol to lysosomes.

**Fig 4:**
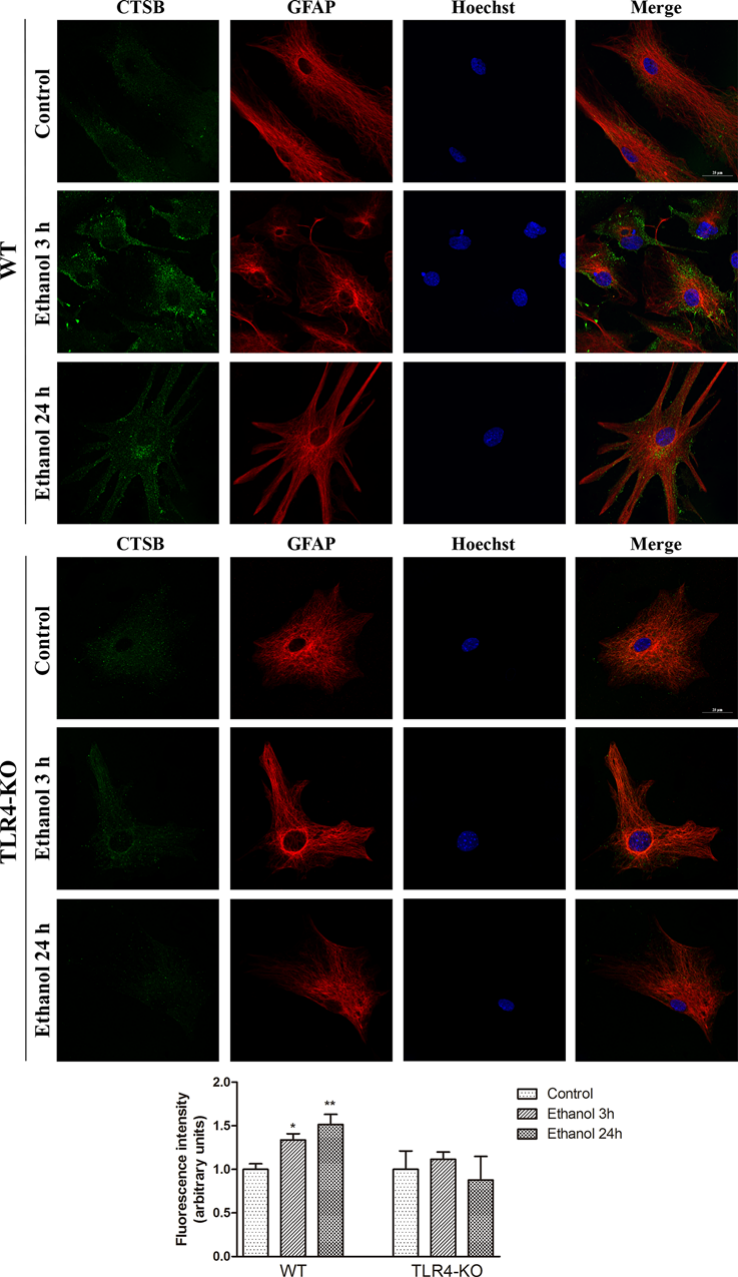
The fluorescence microscopy analysis of cathepsin B in the ethanol-treated WT and TLR4-KO astrocytes. The double-labeling immunofluorescence of cathepsin B and GFAP in the WT and TLR4-KO astrocytes treated with or without ethanol for 3 and 24 h. A representative photomicrograph is shown. Scale bars represent 25 μm. The fluorescence intensity of cathepsin B was quantified using the ImageJ software. Results are the mean ± S.E.M. of data from 4 different fields per condition from 3 different cell cultures. * p < 0.05, ** p < 0.01 vs. the control group.

Finally, in order to assess the correct expression of lysosomal hydrolases and to corroborate the results obtained in Fig 1, we analyzed by fluorescence microscopy the expression of cathepsin B at 3 h and 24 h of ethanol treatment in the WT and TLR4-KO cultured astrocytes. Fig 4 confirms the observations shown for cathepsin B expression in the cellular extracts (Fig 1). Thus ethanol significantly increases cathepsin B expression at 3 h and 24 h after ethanol treatment in the WT astrocytes, whereas no changes are observed in the ethanol-treated TLR4-KO cells (Fig 4).

### Ethanol down-regulates the mTOR pathway in cortical astrocytes: role of TLR4

**Fig 5:**
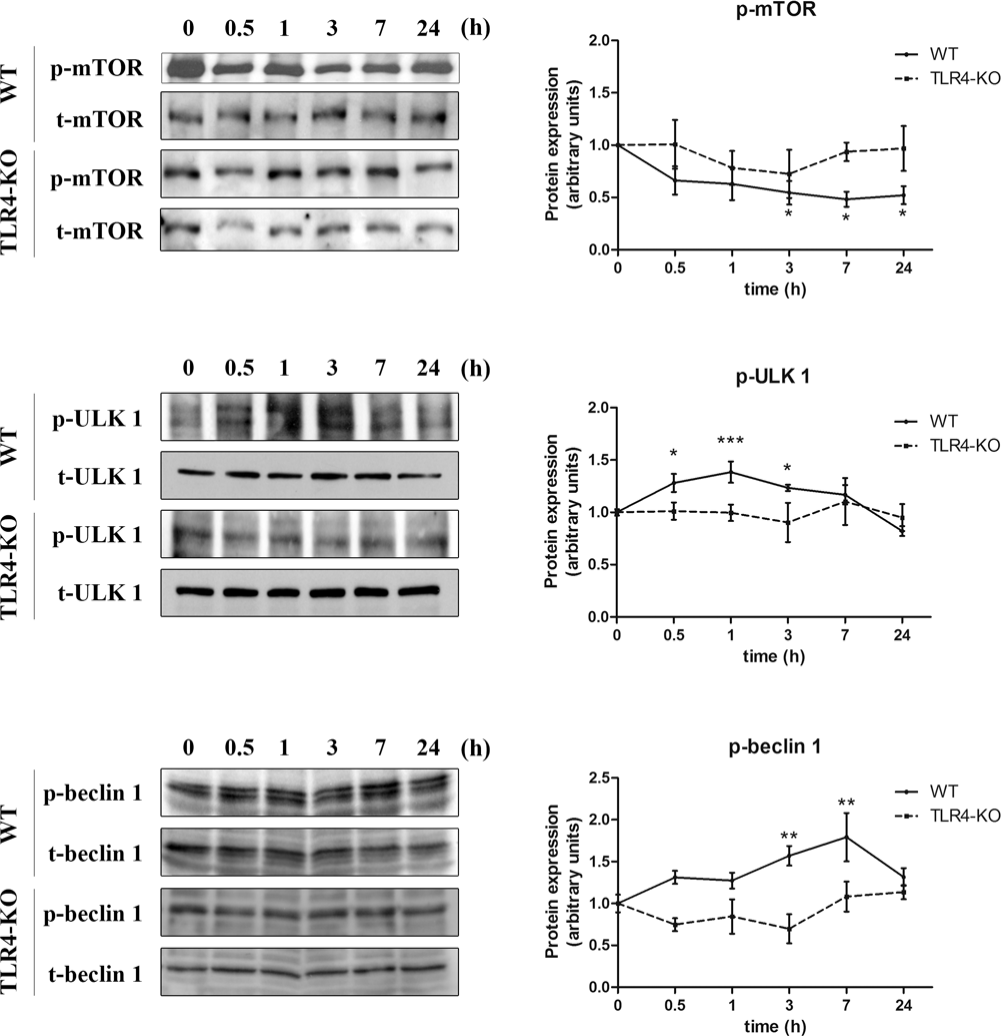
The role of TLR4 in the ethanol-induced down-regulation of the mTOR pathway in cortical astroglial cells. Immunoblot analysis and quantification of p-mTOR, p-ULK1 and p-beclin-1 in the cell extracts of ethanol (50 mM)-treated cells at different time points (0, 0.5, 1, 3, 7 and 24 h). Values represent mean ± SEM, n = 12–15 independent experiments. * p < 0.05, ** p < 0.01, *** p < 0.001 compared with the untreated WT or TLR4-KO value. Blots were stripped, and the total quantities of mTOR, ULK1, and beclin-1 were also assessed. A representative immunoblot of each protein is shown.

To further evaluate the mechanisms involved in the ethanol-induced alterations of the autophagy machinery, we measured the main components of the mTOR pathway. These proteins act in a signaling cascade in which their phosphorylation state regulates their activity. mTOR constitutes the main switch by inhibiting the autophagy process when phosphorylated [22]. As depicted in Fig 5, ethanol down-regulated the phosphorylation of mTOR after 3, 7 and 24 h of treatment in WT astrocytes, although a tendency towards down-regulation is also noticeable as soon as 30 min after ethanol treatment. Downstream of mTOR, Unc-51-like kinase-1 (ULK1, also known as ATG1) favours autophagy when phosphorylated. Our results showed that ethanol up-regulated the phosphorylation of ULK1 very quickly (30 min), which was maintained until 3 h in WT astrocytes (Fig 5). Interestingly, no changes were observed in the phosphorylation levels of both kinases between the ethanol-treated or untreated TLR4-KO glial cells.

Finally, we also studied whether beclin-1 (aka ATG6) was involved in ethanol-induced autophagy activation of astroglial cells, since it is one of the main components of the membrane nucleation complex (beclin-1/Atg14/Vps15/Vps34). Our results demonstrated that ethanol up-regulated the phosphorylation of beclin-1 at 3 h and 7 h in the WT astrocytes, whereas no changes were observed in the ethanol-treated TLR4-KO cells when compared with their control counterparts (Fig 5).

### Effect of autophagy inhibitors in ethanol-induced inflammation and astroglial cell damage

Finally, in order to evaluate the association between the autophagy pathway, and ethanol-induced neuroinflammation and astroglial cell damage, we used several autophagy inhibitors (wortmannin and bafilomycin A1) in ethanol-treated astroglial cells. Whereas wortmannin is a selective inhibitor of PI3K that can inhibit autophagic sequestration, bafilomycin A1 prevents the maturation of autophagic vacuoles by inhibiting fusion between autophagosomes and lysosomes.

**Fig 6:**
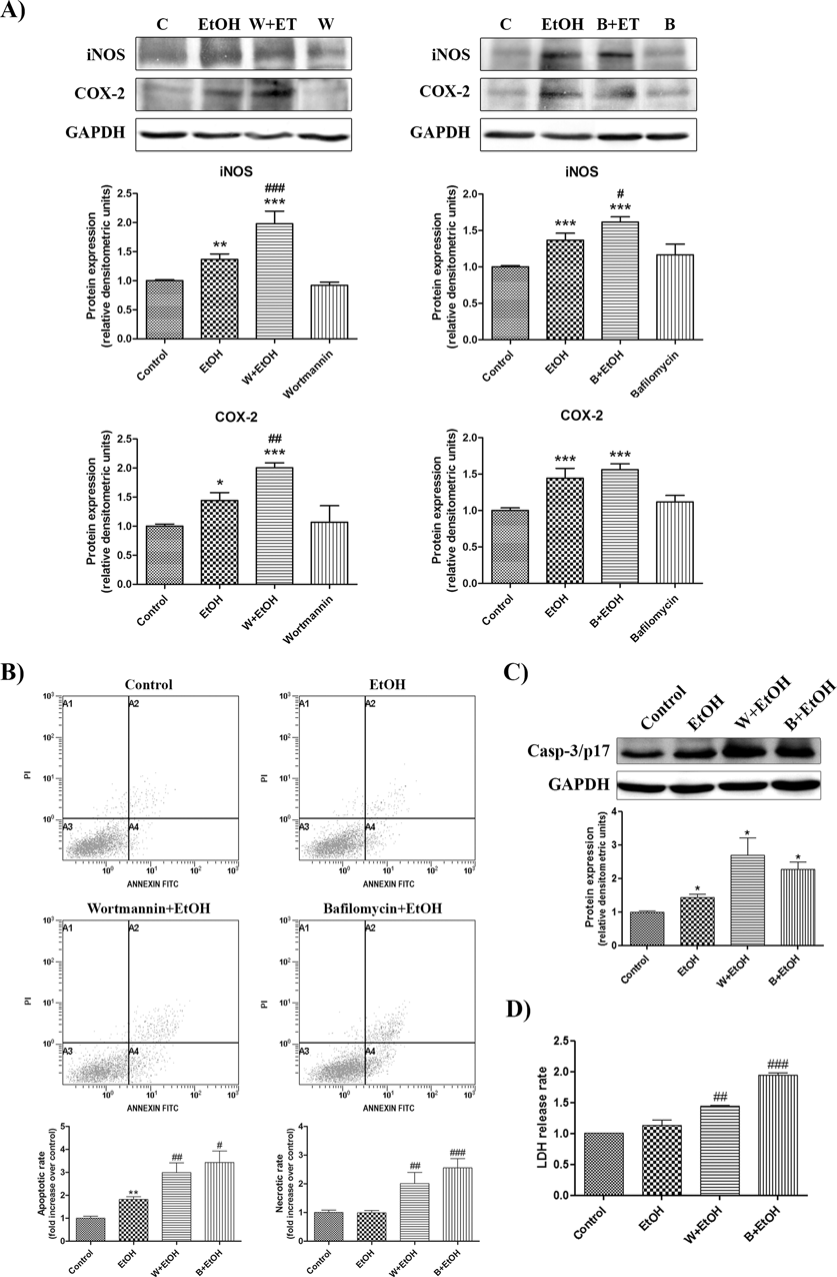
Effect of autophagy inhibitors in ethanol-induced inflammation and astroglial cell damage. Astrocytes from the WT mice were incubated for 24 h either with or without ethanol (50 mM) in the presence or absence of autophagy inhibitors wortmannin (100 nM) and bafilomycin A1 (100 nM). Autophagy inhibitors were incubated in cells 1 h before initiating the 24 h ethanol treatment. (A) Immunoblot analysis and quantification of inflammatory mediators iNOS and COX-2. (B) Apoptotic cell death analysis of cortical astrocytes by flow cytometry. (C) Immunoblot analysis and quantification of the 17kDa active fragment of caspase-3 in cortical astrocytes. (D) LDH activity measured in the supernatant of cortical astrocytes. Data represent mean ± SEM, n = 12–15 independent experiments. * p < 0.05, ** p < 0.01, *** p < 0.001 as compared to the control group, # p < 0.05, ## p < 0.01, ### p < 0.001 as compared to the ethanol-treated group. Blots were stripped, and the total quantity of GAPDH was also assessed. A representative immunoblot of each protein is shown.

Fig 6A shows the changes in the levels of inflammatory mediators iNOS and COX-2 induced by 24 h of ethanol treatment in astrocytes in the presence or absence of autophagic inhibitors. As expected, ethanol treatment increased the levels of both inflammatory mediators. However, wortmannin treatment enhanced the levels of iNOS and COX-2 induced by ethanol in astrocytes, whereas bafilomycin A1 increased only the expression of iNOS as compared to the ethanol-treated astrocytes. No significant changes in the expression of the inflammatory mediators were observed when either inhibitor was added to the cell medium alone.

In addition, we were also interested in evaluating the effects of ethanol on cell death. We assessed the two main different types of cell death, apoptosis and necrosis, using cytometry assays, caspase-3 (17 kDa) and LDH release. We observed that ethanol triggered cell death via apoptosis after 24 h of treatment, as demonstrated by using Annexin V-FITC (Fig 6B) and the 17 kDa active caspase-3-clevage peptide (Fig 6C). Interestingly, the inhibition of autophagy with either wortmannin or bafilomycin A1 enhanced not only the apoptotic process, but also necrosis as compared to the ethanol-treated cells (Fig 6B and 6D), which reveals the importance of autophagy in fighting ethanol toxicity.

### Ethanol down-regulates the autophagy pathway in neurons and triggers necrotic cell death, which is alleviated by the use of rapamycin

**Fig 7:**
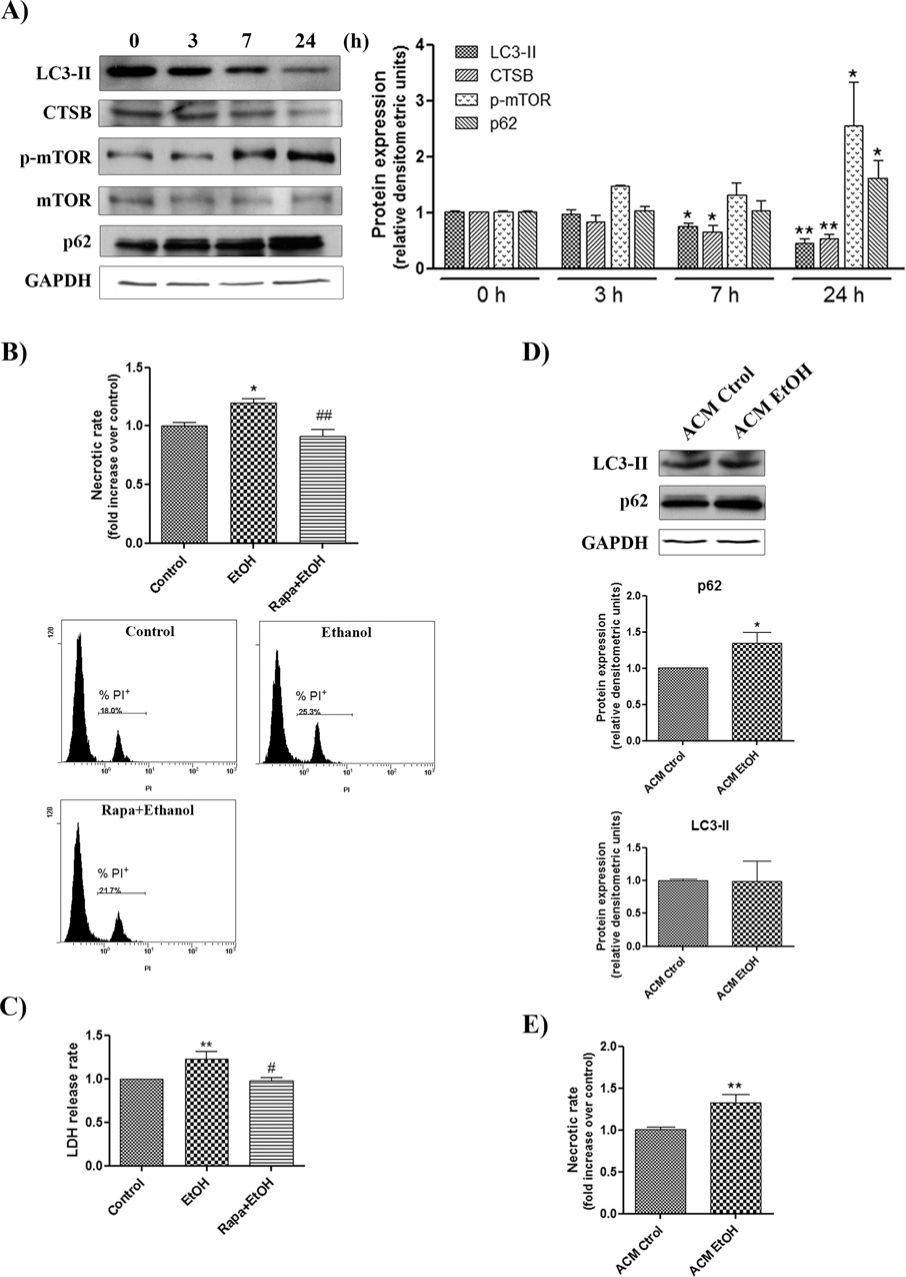
Ethanol down-regulates the autophagy pathway in cortical neurons and also causes necrotic cell death. (A) Immunoblot analysis and quantification of LC3-II, p62, CTSB and p-mTOR in the cell extracts of the ethanol (50 mM)-treated WT neurons at different time points (0, 3, 7 and 24 h). Values represent mean ± SEM, n = 8–10 independent experiments. * p < 0.05, ** p < 0.01 compared with the untreated WT value. Blots were stripped, and the total quantity of GAPDH and mTOR was also assessed. A representative immunoblot of each protein is shown. (B) Autophagy enhancer rapamycin was incubated in the WT neurons for 1 h before initiating the 24 h ethanol treatment. The necrotic cell death analysis of cortical neurons was analyzed by flow cytometry. Data represent mean ± SEM, n = 8–10 independent experiments. * p < 0.05 as compared to the control group, ## p < 0.01 as compared to the ethanol-treated group. (C) LDH activity was measured in the supernatant of cortical neurons, untreated or treated with ethanol and rapamycin (100 nM) plus ethanol. Data represent mean ± SEM, n = 4–5 independent experiments. ** p < 0.01 as compared to the control group, # p < 0.05 as compared to the ethanol-treated group. (D-E) Analysis and quantification of the necrotic cell death (D) and LC3-II and p62 (E) in the extracts of the WT neurons incubated for 1 day with astroglia-conditioned medium (ACM) obtained from the ethanol-treated or non-treated WT astroglial cells (50 mM) for 24 h. Values represent mean ± SEM, n = 6–8 independent experiments. * p < 0.05, ** p < 0.01 compared with the untreated WT value. Blots were stripped, and the total quantity of GAPDH was also assessed. A representative immunoblot of each protein is shown.

Neurons are particularly vulnerable to stress situations or toxic environments [23] and they lack some of the defense mechanisms of the glial cells. Furthermore, the TLR4 expression is low and its role in innate immunity remains controversial [24,25]. Finally, they do not exhibit a strong inflammatory response against toxic compounds, such as ethanol, and relay on the detoxifying capacity of glial cells. We therefore evaluated the potential direct effects of ethanol on the main markers of autophagy, LC3-II, p62, CTSB and p-mTOR, in pure cultures of neurons after 0, 3, 7 and 24 h of ethanol treatment. As shown in Fig 7A, while ethanol treatment lowered the expression levels of LC3-II and CTSB after 7 and 24 h of treatment, p62 and mTOR phosphorylation were up-regulated at 24 h, events that are consistent with autophagy impairment and could be detrimental for neuronal survival. We therefore assessed the potential neuronal cell death after 24 h of ethanol treatment. Analysis by flow cytometry (Fig 7B) and LDH release (Fig 7C) indicated that neurons exhibited an increase in necrotic cell death rate after ethanol treatment, suggesting the importance of autophagy in fighting ethanol-induced neurotoxicity. To test this hypothesis, we added autophagy enhancer rapamycin 1 h prior to the 24-hour ethanol treatment and found that rapamycin diminished the ethanol-induced necrotic death in cortical neurons (Fig 7B and 7C).

We next evaluated whether the production of inflammatory mediators secreted by activated astroglial cells was able to activate the autophagy pathway in neurons. For these experiments, the medium of cortical neurons (at day 5 in culture) was replaced by an astroglia-conditioned medium obtained from those cells treated or not with 50 mM ethanol for 24 h, and the levels of LC3-II and p62, as the two most indicative autophagy markers, were measured. Fig 7D shows that, while no changes were observed in LC3-II expression, the levels of p62 were increased in neurons cultured with the supernatant of astroglial cells treated with ethanol for 24 h when compared to their control counterparts. These data suggest that neurons are not capable of triggering autophagy in the presence of an inflammatory environment produced by astrocytes. Moreover, this inflammatory environment built up by the supernatant of the ethanol-treated WT astroglial cells induced necrotic neuronal death, while no changes were observed in the neurons cultured with the conditioned medium from non-treated astroglial cells (Fig 7E). However, and since the astroglia-conditioned medium might contain some traces of alcohol, we also used neurons incubated with a medium in which ethanol was added at the same concentration found in the conditioned medium of ethanol-treated astroglia, in order to discard the effect of the remaining ethanol. The concentration of ethanol for the supernatant of the astroglia treated with ethanol for 24 h was 4.6 ± 1.6 mM, and it did not induce significant differences in the neuronal cell death as compared with untreated cells (data not shown). Altogether, these results indicate that the inflammatory mediators produced by astroglia do not trigger autophagy in neurons in response to ethanol, and are already capable of inducing neuronal death even without the contribution of ethanol itself, confirming neuronal vulnerability to toxic environments. This was also shown in conditioned medium of ethanol-treated microglial cells [7,26,27], where we also demonstrated that TLR4-KO microglial conditioned medium does not induce neuronal death, indicating the role of this receptor in the release of inflammatory mediators.

## Discussion

Recent evidence indicates the participation of proteolytic processes, such as the ALP, in the regulation of the immune response and the pathology of several neuroinflammatory and neurodegenerative diseases [3]. We demonstrated that ethanol is capable of triggering the expression of cytokines and other inflammatory mediators through the activation of innate immune receptor TLR4, which ultimately leads to brain damage and neurodegeneration in chronic ethanol-drinking mice [7,8]. We further showed that chronic ethanol intake alters the proteolytic pathways *in vivo* in mice brain and that this process is mediated by TLR4 signaling [5]. However, whether an acute dose differently affects the autophagic pathway in astroglial cells or neurons, and which is the role of the proteolytic mechanisms in modulating neural cell death, remains elusive. We herein show for the first time that the induction of autophagy via TLR4 constitutes a protective mechanism triggered by astrocytes to fight ethanol-induced toxicity. We also provide evidence that ethanol has a more harmful effect on neurons, which express low levels of TLR4 response and showed reduced autophagy.

Contradictory results on the effect of ethanol on autophagy have been reported. Whereas some studies have suggested that ethanol impairs the autophagic process in the liver [28,29] or in several immortalized cell lines [10], other authors have reported that autophagy could serve as a protective cellular mechanism against alcohol-induced tissue injury [12,30–32]. Indeed, our results reveal that ethanol affects autophagy in a different way that is dose- and treatment-dependent (acute *vs* chronic). Our previous findings show that chronic ethanol consumption impairs autophagy, with a reduction in several ATG protein levels and an up-regulation of mTOR phosphorylation [5]. However, we herein demonstrate that a single acute ethanol dose is capable of inducing the ALP in mice astrocytes by up-regulating the main ATG proteins, such as ATG12, LC3-II/ATG8 or beclin-1/ATG6, at different time points. These three proteins are known to be essential in the induction of autophagy as they participate in autophagosome formation [33]: beclin-1 is involved in the nucleation process, whereas ATG12 and LC3-II take part in the conjugation pathway to produce the phagophore elongation. LC3-II interacts with p62 to induce a type of autophagy known as “selective autophagy”, since it recognizes ubiquitinated proteins and diverges them from the ubiquitin-proteasome system to the autophagy pathway. When autophagy is induced, the basal pool of p62 protein interacts with LC3-II to then become degraded within the autolysosome [16], and therefore p62 levels decline. Accordingly, we found a down-regulation of p62 after ethanol treatment, which suggests that ethanol induces autophagy in mice astrocytes. Ours results also agree with recent findings, which have shown that ethanol up-regulates the expression of LC3-II and beclin-1 and the degradation of p62 in the developing brain and in SH-SY5Y neuroblastoma cells [30].

ATG proteins are mainly controlled by the mTOR pathway, which serves as a master regulator of autophagy, by inhibiting it when phosphorylated [34]. In fact, mTOR directly controls the activity of the complex formed by ULK1/ATG1, ATG12 and FIP200, which constitutes the first inducer of the membrane nucleation process of the phagophore. We show that an acute ethanol dose is capable of inhibiting mTOR, which in turn elicits ULK1 activation through its phosphorylation. Inhibition of the mTOR pathway by ethanol has also been suggested [12,35].

The results of the present study support the idea that an acute ethanol dose increases the autophagic flux in astrocytes. In line with this hypothesis, our microscopy studies demonstrated that a 24-hour ethanol treatment induced the overexpression of autophagic vacuoles and up-regulated the production of lysosomes, the acidic organelles that fuse with autophagosomes to form the autolysosomes, these being the final subcellular compartments in which the degradation process takes place. Accordingly, the western blot and confocal microscopy studies revealed that ethanol increased the expression of cathepsin B, one of the main lysosomal hydrolases. Unexpectedly, lysosomes showed a slight increase (basification) in pH after 3 hours of ethanol treatment. This last observation suggests that some form of damage is caused by ethanol, although the engulfment of pH alkaline cytosolic material by active lysosomes could also be possible [21]. Altogether, our data strongly suggest that ethanol induces autophagy in mouse astrocytes by inhibiting the mTOR pathway, and hence promoting the expression of ATG proteins, which leads to an increased autophagic flux, as characterized by electron microscopy. However, why ethanol has opposing effects after acute or chronic treatment remains to be elucidated and deserves further investigation. Some authors pointed recently to this duality of ethanol and suggest that, although the induction of autophagy is beneficial against toxic insults, a persistent and overactivated autophagic response could induce cellular and organelle damage, which would result ultimately in autophagic machinery failure, as suggested by Ding *et al*. [36].

A relevant finding of the present study is the demonstration that only minor changes are detected in astrocytes from TLR4-KO mice. Our previous studies have discovered the implication of TLR4 activation in the induction of inflammatory cytokines and inflammatory mediators, which ultimately lead to brain damage and neurodegeneration [7,26,27]. The present results suggest that TLR4 signaling is also involved in the modulation of the autophagic process in glial cells, since we observed neither an overexpression of autophagic proteins nor an induction of autophagosome formation in TLR4-KO astrocytes. Not surprisingly, TLRs have been shown to promote autophagy, and this induction seems to depend on the activation of beclin-1 by MyD88 and TRIF, the main two effectors of the TLR4 signaling cascade [37–39]. In addition, the TRIF pathway seems to activate the formation of autophagosomes before fusing with lysosomes [39].

Autophagy is a tightly regulated process that has been shown to respond rapidly in stress situations, including starvation, organelle damage, hypoxia or oxidative stress [3,40]. However, the role of autophagy in the regulation of cell death *vs* cell survival is controversial. It is noteworthy that the induction of autophagy typically occurs in stress situations characterized by a hostile environment, such as bacterial infection, where the cell has to decide whether to fight the toxic element or undergo apoptosis. Therefore, on the one hand, autophagy has been linked to cell death either directly by the so-called autophagic cell death (type II) or through an interaction with the pathways related to apoptotic (type I) or necrotic cell death (type III), such as Bcl-2 [41,42]. On the other hand, several studies have proposed that autophagy is an important self-defense response against toxic insults [43,44]. This is especially important in the context of neurodegenerative diseases, such as Alzheimer’s, Parkinson’s or Huntington’s disease, where induction of autophagy has been shown to alleviate the pathological phenotype [45,46]. Our results support the hypothesis of autophagy acting as a protective mechanism against ethanol toxicity, since it protects astrocytes against ethanol-induced neuroinflammation and cell death, at least after an acute ethanol dose. In fact, the administration of autophagy inhibitors, such as wortmannin or bafilomycin A1, further increases the production of inflammatory mediators, such as iNOS and COX-2, triggered by ethanol. These two compounds also increase the apoptotic rate and trigger necrosis in the WT astrocytes.

The present findings further demonstrate the differential role of autophagy against ethanol toxicity in neurons and astrocytes in primary culture. Neurons are post-mitotic cells and their ability to cope with toxic stimuli may differ from that of other cell types, such as neuroblastoma SH-SY5Y cells [30] or glial cells, which can proliferate and regenerate [47]. Indeed, we show that the effect of ethanol on autophagy was the opposite to that observed in astrocytes, as demonstrated by ethanol down-regulating LC3-II, cathepsin B and increasing the expression of p62 and p-mTOR in neurons, thus inhibiting autophagy. This impairment of the autophagic process by ethanol in neurons correlates with the triggering of necrotic neuronal death after 24 hours of treatment, hence supporting the hypothesis of a protective role of autophagy. Interestingly, the necrotic process could be prevented by administering the autophagy inducer rapamycin. Conversely, Chen *et al*. reported that ethanol induces the autophagy pathway in neuroblastoma SH-SY5Y cells [30]. One possible explanation for this divergence could rely in the different characteristics of these neuroblastoma cells and those of more physiologically relevant cells, such as cortical neurons in primary culture, which lack the self-renewal potential of immortalized cells.

Finally, although the differential effects of ethanol on autophagy in cortical neurons and glial cells in primary culture are unclear, several possibilities can be suggested: 1) neurons seem to have less capability than astrocytes to cope with stress and exhibit a poorer autophagic response [48]; 2) neurons lack the TLR4 response *vs* the amplified TLR4 signaling response in glial cells [25]; 3) neurons typically rely on glial cells for support and for their protection. Nevertheless, it is to be noted that even in the context of a protective autophagic response against ethanol toxicity, the inflammatory environment produced by astrocytes is already toxic for neurons, as demonstrated by the use of astrocyte-conditioned media in neuronal cultures. Indeed, our previous studies have shown that ethanol, as LPS, is capable to induce TLR4 signaling in glial cells [13,27], leading to the production of cytokines in the medium which can cause neuronal death [27].

## Conclusions

Taken together, these results demonstrate that ethanol triggers autophagy in mouse cortical astrocytes and that this induction is mediated through the innate immune receptor TLR4. Furthermore, we show that autophagy constitutes a self-defense and pro-survival mechanism triggered by astrocytes to fight ethanol-induced toxicity, and that it is capable of alleviating the inflammatory process and preventing cell death. Conversely, ethanol down-regulates autophagy in neurons, which hence become more exposed to cellular damage and cell death. Finally, neuron-glial interactions are critical for the normal function and viability of neurons, and should be further examined in the context of neuroprotective mechanisms such as autophagy.

## Supporting Information

S1 Fig TLR4 participates in the ethanol-induced overexpression of several autophagic proteins in cortical microglial cells. TLR4 participates in the ethanol-induced overexpression of several autophagic proteins in cortical microglial cells. Primary mouse cultures of cortical microglial cells were prepared as previously described (Fernandez-Lizarbe et al., 2009). Immunoblot analysis and quantification of ATG5, ATG12, cathepsin B, LC3-II and p62 in cell extracts of ethanol (50 mM)-treated cells at different time points (0, 0.5, 1, 3, 7 and 24 h). Values represent mean ± SEM, n = 12–15 independent experiments. * p < 0.05, ** p < 0.01, *** p < 0.001 compared with the untreated WT or TLR4-KO value. Blots were stripped, and the total quantity of GAPDH was also assessed. A representative immunoblot of each protein is shown.
(TIF)

S1 Table Basal level of the proteins analyzed in WT and TLR4-KO astrocytes. (DOC)
