# Supplementary figures and images for: Autophagy Constitutes a Protective Mechanism against Ethanol Toxicity in Mouse Astrocytes and Neurons
Source: PLoS One. 2016 Apr 12;11(4):e0153097. doi: 10.1371/journal.pone.0153097 (PMC4829237; doi:10.1371/journal.pone.0153097)

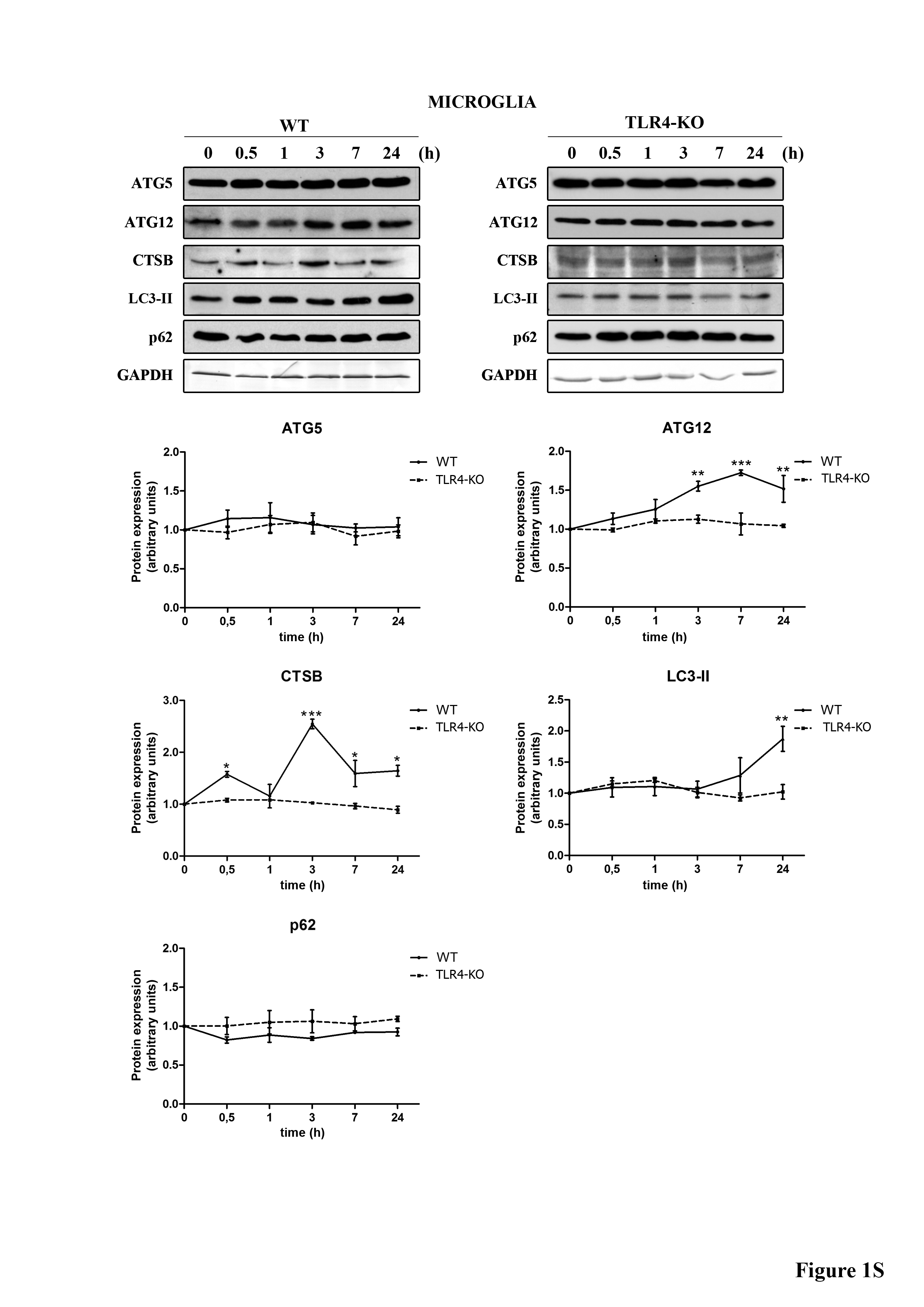

Supplement: S1 Fig — TLR4 participates in the ethanol-induced overexpression of several autophagic proteins in cortical microglial cells. Primary mouse cultures of cortical microglial cells were prepared as previously described (Fernandez-Lizarbe et al., 2009). Immunoblot analysis and quantification of ATG5, ATG12, cathepsin B, LC3-II and p62 in cell extracts of ethanol (50 mM)-treated cells at different time points (0, 0.5, 1, 3, 7 and 24 h). Values represent mean ± SEM, n = 12–15 independent experiments. * p < 0.05, ** p < 0.01, *** p < 0.001 compared with the untreated WT or TLR4-KO value. Blots were stripped, and the total quantity of GAPDH was also assessed. A representative immunoblot of each protein is shown. (TIF) [file pone.0153097.s001.tif]
