# Supplementary material for: Autophagy Constitutes a Protective Mechanism against Ethanol Toxicity in Mouse Astrocytes and Neurons
Source: PLoS One. 2016 Apr 12;11(4):e0153097. doi: 10.1371/journal.pone.0153097 (PMC4829237; doi:10.1371/journal.pone.0153097)
Supplement: S1 Table — (DOC) [file pone.0153097.s002.doc]

**Table 1S.** Basal level of the proteins analyzed in WT and TLR4-KO astrocytes.

|  |  | | Mean ± SEM | p |
| --- | --- | --- | --- | --- |
| Atg5 | Control WT  Control TLR4-KO | 1.00 ± 0.11  1.14 ± 0.14 | | 0.48 |
| Atg12 | Control WT  Control TLR4-KO | 1.00 ± 0.12  1.06 ± 0.10 | | 0.77 |
| CTSB | Control WT  Control TLR4-KO | 1.00 ± 0.08  0.74 ± 0.06 | | 0.12 |
| LC3-II | Control WT  Control TLR4-KO | 1.00 ± 0.11  0.84 ± 0.04 | | 0.31 |
| P62 | Control WT  Control TLR4-KO | 1.00 ± 0.19  1.38 ± 0.18 | | 0.23 |
| p-mTOR | Control WT  Control TLR4-KO | 1.00 ± 0.12  1.13 ± 0.12 | | 0.56 |
| p-ULK1 | Control WT  Control TLR4-KO | 1.00 ± 0.26  1.09 ± 0.04 | | 0.79 |
| p-beclin 1 | Control WT  Control TLR4-KO | 1.00 ± 0.18  1.31 ± 0.11 | | 0.39 |

No significant differences were observed in the basal levels of the analyzed proteins in WT and TLR4-KO astrocytes. Data represent mean ± SEM, n = 3-4 independent experiments. All data were analyzed using an unpaired Student’s t-test.
